# Murine patellar tendon transplantation requires transosseous cerclage augmentation — development of a transplantation model for investigation of systemic and local drivers to healing

**DOI:** 10.1186/s13018-019-1475-4

**Published:** 2019-12-02

**Authors:** Borys Frankewycz, Daniel Cimino, Nelly Andarawis-Puri

**Affiliations:** 1 Sibley School of Mechanical and Aerospace Engineering, College of Engineering, Cornell University, Ithaca, NY USA; 2 Department of Trauma Surgery, Regensburg University Medical Center, Regensburg, Germany; 3 Department of Clinical Sciences, Cornell University College of Veterinary Medicine, Cornell University, Ithaca, NY USA; 4 Hospital of Special Surgery, New York, NY USA

**Keywords:** Murine patellar tendon, Patellar tendon transplantation, Tendon regeneration

## Abstract

**Background:**

Tendon injuries are common musculoskeletal injuries that heal with scar tissue formation, often achieving reduced biomechanical and functional properties. The murine patellar tendon is a research tool that holds potential for investigating tendon healing and can be useful for exploring therapeutic strategies. Since healing is a complex process that results from the collaboration between the systemic and local tissue environment, a murine tendon transplantation model that can be applied to transgenic mice and genetic mutants would allow isolation of systemic versus local tendon factors in driving effective tendon healing. Preliminary studies have shown that transplantation with simple tendon sutures results in a proximalization of the patellar bone due to the involuntary quadriceps muscle force leading to tearing of the graft and failure of the knee extensor mechanism. To avoid this elongation of the graft, two cerclage techniques for murine patellar tendon transplantation were introduced and validated.

**Methods:**

Three developed surgical techniques (no-cerclage-augmentation (NCA)), transfascial suture cerclage with encirclement of the patellar tendon (TFSC), and dual-cerclage-augmentation with a transosseous bone-to-bone cerclage through the patella bone and an additional musculotendinous cerclage (DCA)) were compared at 4 and 8 weeks macroscopically in regards to graft continuity, cerclage integrity, gap formation, and radiologically by measuring the patello-tibial distance and using a patella bone position grading system.

**Results:**

The NCA group showed complete failure at 5–7 days after surgery. The TFSC has led to 69% functional failure of the cerclage. In contrast, the DCA with a has led to 78% success with improvement in patellar bone position and a similar patello-tibial distance to the naïve contralateral murine knees over the time period of 8 weeks.

**Conclusions:**

This study shows that a bone-to-bone cerclage is necessary to maintain a desired graft length in murine patellar tendon models. This surgery technique can serve for future graft trans- and implantations in the murine patellar tendon.

## Background

Tendon injuries are common debilitating and costly musculoskeletal injuries [1]. Tendons ineffectively heal through scar formation leading to decreased biomechanical properties [2, 3]. Both the systemic environment and the local tendon environment likely encompass aspects that should be harnessed to develop effective therapeutics. Developing a tendon transplant model that can be used with the widely available genetic mutants and inbred strains will be a valuable tool towards the advancement of effective therapeutics.

The murine patellar tendon (PT) is commonly used in musculoskeletal research. The PT is unique in that it has an osseous insertion on both ends, connecting the patellar bone (PB) to its tibial insertion [4–6]. The flat shape of the PT makes it conducive for use as a full-thickness central punch model [7, 8]. Compared to other murine tendon injury models, the PT central punch model has vastly improved on consistency and reproducibility [9].

Tendon transplantation and graft implantation are common techniques to investigate tendon healing characteristics. Allograft and xenograft transplantation allows the exploration of inter-strain and interspecies differences in biomechanical and biological aspects [10, 11]. Additionally, since healing is a complex process that results from collaboration between the systemic and local tissue environment, a murine tendon transplantation model that can be applied to transgenic mice and genetic mutants, would allow isolation of systemic versus local tendon factors in driving effective tendon healing [7, 12, 13]. Moreover, the field of translational tissue engineering requires implantational or transplantational techniques of grafts to achieve successful in-vivo results [14, 15]. Although a murine PT-transplantation model has been published, neither technical details nor success rates have been reported [16].

A major challenge to tendon implantation is the stump-site adaptation. Tendon healing requires time; therefore, postoperative inhibition of the corresponding muscle is required to allow the formation of a stable continuity of the tendon tissue [17]. However, a complete inhibition cannot be achieved due to the involuntary muscle tension, especially in animals. On the other hand, a tensile stimulus is required for the formation and maturation of tendinous tissue [18–20]. Therefore, a balance of immediate tension and immobilization after surgery is crucial.

## Materials and methods

The purpose of this study is to establish a suitable surgery technique for murine patellar tendon implantation that takes into account these considerations. Three surgery techniques will be compared in regard to their postoperative patella position.

### Animal model and surgical procedure

The dual-cerclage-augmentation (DCA) technique is described below. Please refer to supplemental data for the preliminary, no-cerclage-augmentation (Additional file 1) and transfascial suture cerclage (Additional file 2), techniques. All procedures were approved by the Institutional Animal Care and Use Committee (Cornell University, Office of Research Integrity and Assurance, protocol number 2015–0120 and appendant Refinement protocols).

**Fig. 1:**
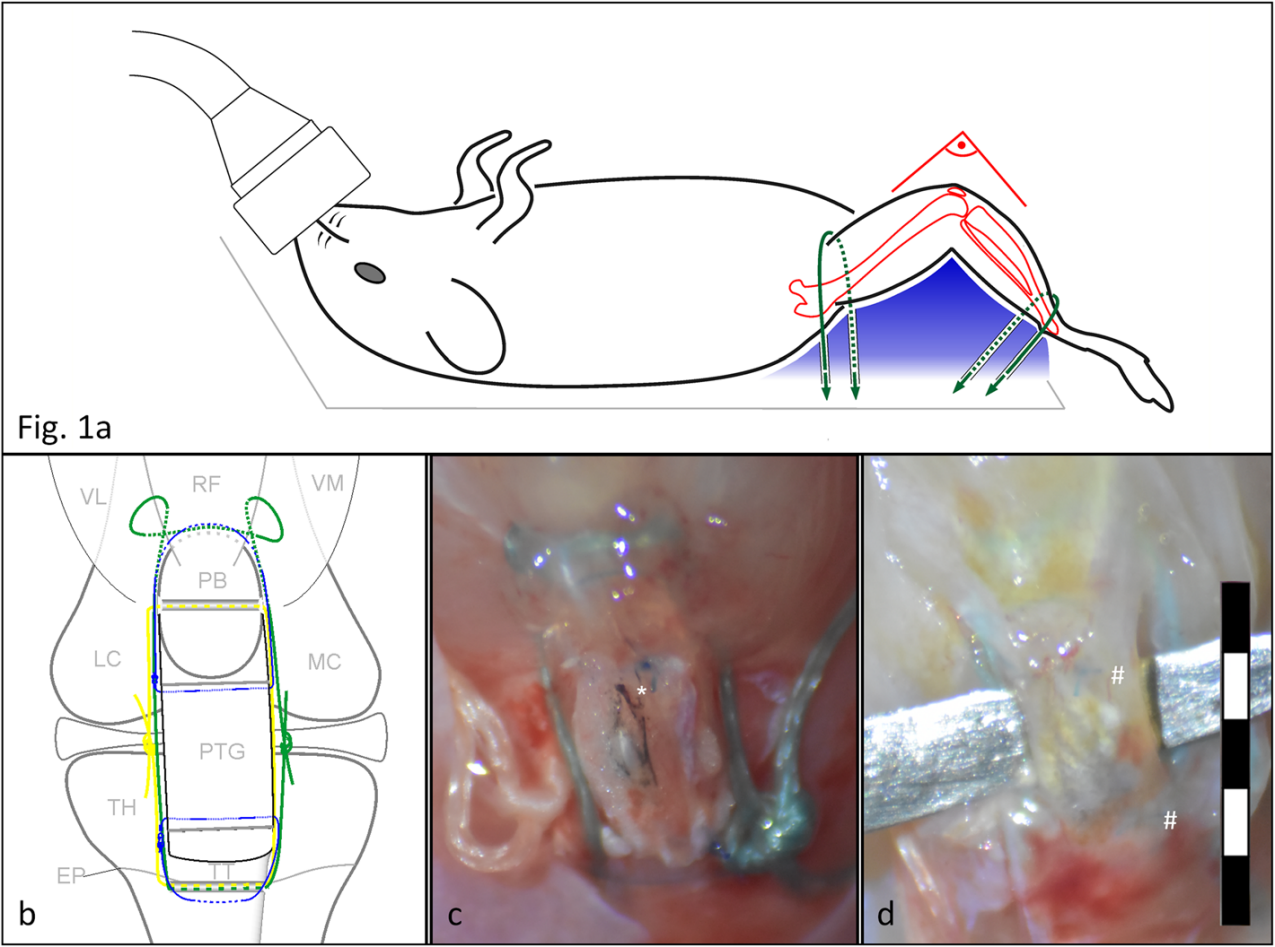
**a** Animal position during surgery: to standardize the knee position during the surgery, mice were placed on a custom made fixture (Plaster of Paris) with a pre-shaped body groove and a support rim for the right knee. Both ankle and groin were strapped down with 0.6 mm cords (green) to align the thigh and calf into the support rim (blue), creating a 90° knee angle position. **b** Schematic of the surgery model: PB = patellar bone, VL = vastus lateralis, RF = rectus femoris, VM = vastus medialis, FC = femoral condyles, TT = tibial tuberosity, TH = tibial head, EP = epiphyseal plate. The corresponding post-operative macro image (**c**) shows the graft in-vivo: the tibio-patellar cerclage (TPC, white suture, yellow in the schematic) is a bone-to-bone cerclage whose function is to directly bypass the function of the patellar tendon, thus to transfer the tensile force of the quad-muscle onto the joint (functionally: knee extension). Additionally, a tibio-musculotendinous cerclage (TMC, green suture) facilitates the construct and due to its more proximal insertion, framing the patellar bone, it is meant to reduce the luxation of the patellar bone. The two graft sutures (blue sutures) are stitched through the graft and the corresponding tendon stump to keep the graft in place. The long free suture ends of both cerclages are placed subcutaneously far away from the construct to reduce tissue granulation. (* indicates the India ink which was used to mark the central full-thickness defect used for a different study). **d** Macro image after sacrifice: the graft is elevated on a backing, the whole construct is covered with a layer of connective scar tissue. Both graft suture knots are visible underneath (#) and the graft shows proximal and distal continuity within the patello-tibial construct


Fifteen- to 16-week-old male C57BL/6 J and MRL/MpJ mice were purchased from The Jackson Laboratory (Bar Harbor, ME). Mice were anesthetized with isoflurane and received preoperative 0.06 mg/kg buprenorphine subcutaneously. The fur of the right hindlimb was removed using Nair and then rinsed. The anesthetized mouse was placed on a custom made a fixture with a 90° knee support for the right limb. The ankle and groin were restrained onto the table to keep the knee in the same position during the entire surgery (Fig. 1a). Surgeries were performed under tip-sterile conditions. A schematic of the surgery model is shown in Fig. 1b. A video with all surgery steps is attached in Additional file 3.

**Additional file 3.** Murine patellar tendon transplantation video. Commented macroscopic video of the surgical steps of the DCA method in HD.


### Surgical approach

Following a medial 1 cm incision between the PT and saphenous vein, the skin was retracted laterally to expose the knee joint. After fasciectomy, two adjacent 0.2 mm drill holes were drilled underneath the tibial tuberosity level with the epiphyseal growth plate. Through these holes, two cerclage sutures were pulled through. A white 5–0 Mersilene suture (type R690; Ethicon, Bridgewater, NJ) was used for a bone-to-bone cerclage (tibio-patellar cerclage (TPC)) and a green 5–0 Ethibond Excel suture (type X727H; Ethicon, Bridgewater, NJ) was used as a bone-to-quad-tendon cerclage (tibio-musculotendinous cerclage, TMC). Both needle-less ends of the two sutures were pulled through the drill hole using a fine beading needle (BeadSmith, Carteret, NJ).

#### Patellar tendon explantation

A bilateral incision was made through the medial and lateral retinaculum and a customized 1.6 mm backing was placed underneath the tendon. For graft fixation, a double-armed 9–0 polypropylene graft suture was stitched four times through the proximal and distal portion of the tendon, respectively (type P90062; Visionary Medical Supplies, Madison, WI). All four suture ends were lifted and clipped to a helping hands device (QuadHands, Alphidia, Charleston, SC). The tendon was sharply transected on both ends in close proximity to the PB and tuberositas tibiae and lifted. The tendon was placed on an acrylic sheet with light tension on the distal and proximal sutures to spread and flatten the tendon. For the purposes of our studies, a 0.75 mm biopsy punch (Robbins Instruments, Chatham, NJ), coated with India ink for defect tracing (Higgins, Chartpak, Leeds, MA), was used to create a central, full-thickness defect. Meanwhile, both cerclages were put in place to avoid postoperative patellar bone proximalization. The TPC was pulled through a 0.2 mm central medio-lateral drill hole made in the patella bone. The TMC was stitched in a modified Kirchmeyr-Kessler manner through the musculotendinous junction of the quad tendon. Both cerclages were knotted firmly with a 15–20% shortening of original tendon stumps distance. For allotransplantation, these steps were performed consecutively on a C57BL/6 J and a MRL/MpJ mice, and tendons were switched for replantation.

#### Implantation

The transplant was placed in the stump-to-stump gap and the preplaced graft suture was stitched back through the lateral retinaculum, around the PB/tuberositas tibiae, through the medial retinaculum and then knotted to partial tightness to avoid tendon displacement and suture pull-out. The skin was closed using a 6–0 Prolene suture (Ethicon, Bridgewater, NJ) (Fig. 1c). The animals were placed on a warm surface and 0.06 mg/kg of buprenorphine was administered daily for 2 days. Sagittal X-rays were taken weekly to ensure an intact PB.

### Clinical evaluation

Once a week, the animals were clinically evaluated regarding their hindlimb usage and the ability of full weight bearing on the operated knee. The criterion “hindlimb usage” was graded into “abundant usage,” “limping,” and “running with full hindlimb usage and symmetrical gait.” To fulfill the criterion “full weight-bearing,” the animals had to stand symmetrically on their operated hindlimb at the cage edge with a fully loaded, extended knee.

### Macroscopic evaluation

After 4 or 8 weeks, mice were sacrificed using CO_2_ following institutional standards and PTs were harvested (Fig. 1d). These time points were chosen for evaluation as they are sufficiently long to evaluate the structural changes during tendon healing [21, 22]. The graft was evaluated and photographed under a dissection microscope for graft continuity, cerclage integrity, and gap formation at the two stump sites.

### Radiographic assessment

**Fig. 2:**
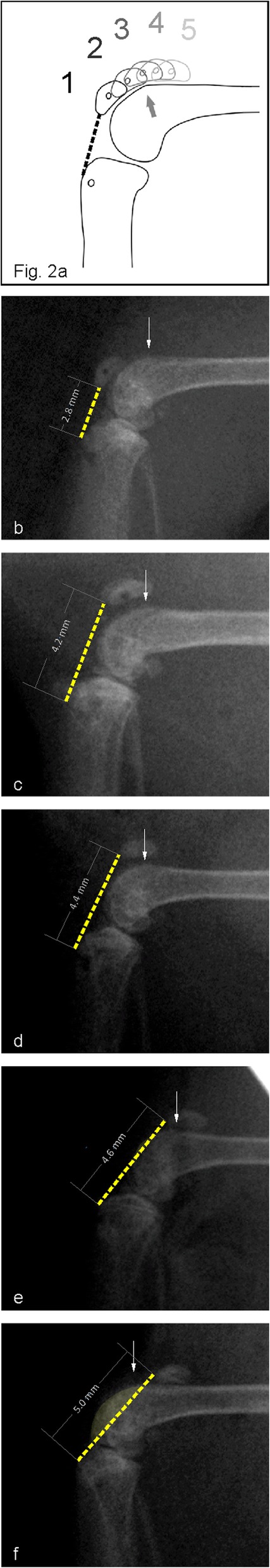
**a** Scheme of the patellar bone position (PBP) grading system: the patella bone position is evaluated relative to the supraconylar spur (arrows). Grade 1 (**b**) is defined as a fully distally located PB to the spur, grade 2 (**c**) with most of the PB length being distal to the spur, grade 3 (**d**) with half of the PB being on top of the spur, grade 4 (**e**) with most of the PB length being proximal to the spur and grade 5 (**f**) with the whole PB located proximal to the spur. The representative X-ray images also show an increase of the measured patello-tibial distance (PTD). These were measured from the distal pole of the PB to the tibial tuberosity (white lines). The dashed lines illustrate the probable graft plane. At grades 4 and 5 that line crosses the condyles (marked area), implying that the graft is bent over the patellofemoral surface of the femur

Sagittal radiographic images were captured of each animal’s stifle at weeks 4 and 8 post-surgery in a standardized 90° flexion position using a dental X-ray machine (Progeny VetVision DC, Midmark, Kettering, OH). Image analysis was performed with ImageJ (Ver. 1.51n, NIH, Bethesda, MD). A patella bone position grading system (PBP) was established: the patella position relative to the supracondylar spur was graded on a scale from 1 to 5 (Fig. 2a). Additionally, the patello-tibial distance (PTD) was measured on each radiograph using the distal end of the patellar bone and the tibial tuberosity as landmarks. Control contralateral tendons (*n* = 31) were similarly graded.

### Statistical analysis

Data is presented as mean ± SD; [range]. After confirming normal distribution using a KS normality test, *t* tests were used to compare PTDs and PBP at the two different time points. A one-way ANOVA was used to compare the PTDs and PBPs of the three surgery methods and to the contralateral limbs with Bonferroni post hoc tests. Kendall’s τ-b rank correlation coefficient was used to correlate values of clinical and radiological parameters. Values of *p* < 0.05 were considered significant.

## Results

Out of 194 animals (101 C57BL/6 J and 93 MRL/MpJ), 9 had an intraoperative patella breakage, 26 had a postoperative patellar fracture with complete distraction of the two patellar poles (after 20 ± 13 days) and 7 showed a postoperative patellar luxation (after 14 ± 8 days). The overall surgery-related failure rate was 22% and these animals were excluded from further evaluation. Another 23 animals were sacrificed on the 7th postoperative day for a different study and 21 were excluded because of other technical issues.

### Clinical evaluation

The animals reached full hindlimb usage at 2 ± 1; [1–6] weeks and full weight-bearing at 2 ± 0.7; [1–6] weeks, respectively. In particular, 60% achieved full usage at week 2, 90% at 3, 99% at week 4 and all of the animals at week 6. Regarding weight-bearing, 92% loaded fully at week 2, 98% at week 3 and 100% at week 5. All animals of both time groups achieved full hindlimb usage and full weight-bearing before sacrifice.

### Macroscopic evaluation

All evaluated specimen showed full continuity of the tendon graft. Three appeared buckled (3%), two were laterized (2%) and one was elongated (1%). Regarding the proximal stump union, 25 specimens (23%) showed formation of a gap between stump and graft, which was filled with scar tissue (*n* = 6: [≤ 0.1] mm, *n* = 7: [0.2–0.5] mm, *n* = 12: [0.6–1.0] mm). On the distal site 12 grafts (11%) showed gap formation (*n* = 3: [≤ 1] mm, *n* = 7: [0.2–0.5] mm, *n* = 2: [0.6–1.0] mm). From these animals three had gap formations on both sides of the graft. In regard to the cerclage continuity, six had a torn TPC and two a torn TMC. Five more TMCs were loose or elongated, but with an intact TPC. A weak correlation was found between cerclage failure and graft elongation (τ-b = 0.342 for TMC and 0.399 for TPC, *p* < 0.0001, respectively) as well as between TPC failure and a proximal gap formation (τ-b = 0.250, *p* = 0.01).

### Radiographic assessment

**Fig. 3:**
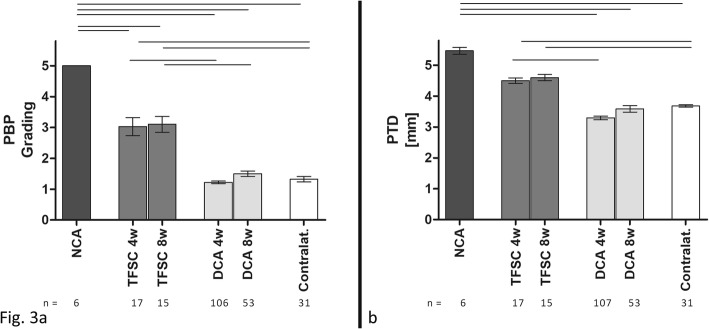
Patellar bone position (PBP) grading (**a**): the dual-cerclage-augmentation technique (DCA) shows a significantly improved distalization of the patella bone position compared to transplant without any cerclage (NCA) or a simple cerclage without a transosseous patella fixation (TFSC). Even though the TFSC technique had better stabilization than NCA, the differences were still significant to the normal, contralateral values. **b** The measured patello-tibial distance (PTD) was comparable to the contralateral tendon, resembling a sufficient counterforce to the quadriceps muscle, whereas the NCA and TFSC groups were significantly elongated. (One-way ANOVA, rods indicating significant differences with *p* < 0.0001)

No differences were found between the two mice strains in regards of PTDs or PBPs; therefore, their values were pooled for further analysis. PTD was 3.2 ± 0.64;[2.1–4.9] mm at 4 weeks and 3.5 ± 0.74;[2.3–5.4] mm at 8 weeks (*p* = 0.0015). PBP was 1.0 ± 0.42;[1.0–3.0] at 4 weeks and 1.0 ± 0.64;[1.0–4.0] mm at 8 weeks (*p* = 0.0016). Just one sample had PBP grade 3. Significant differences were found in regards to the PTD and PBP when comparing the DCA technique with the previously described NCA and TFSC techniques (Additional file 1 and Additional file 2), (Fig. 3). Additionally, a moderate correlation was found between PTDs and PBPs at both time points (τ-b = 0.613, *p* < 0.0001 at 4 weeks and τ-b = 0.628, *p* < 0.0001 at 8 weeks). A weak correlation was found between the failure of the TPC and the PBPs (τ-b = 0.349, *p* < 0.0001 at 4 weeks and τ-b = 0.355, *p* = 0.009 at 8 weeks).

## Discussion

This study established a feasible model of patellar tendon transplantation in mice that can be used with the widely available genetic mutants and inbred strains to determine systemic and local factors that are essential to effective tendon healing. Tendon transplantation requires consideration of biomechanical and technical aspects. First, without augmentation, postoperative immobilization of the joint complex would be necessary to allow the tendon stumps to heal in continuity and by that to create a stable bioactive mechano-transductive unit [23]. However, in animals partial immobilization would require elaborate mechanical restraint and weight-bearing instructions will not be complied. Second, a full-size transection of the tendon, if not stabilized, leads to a retraction of the tendon stumps due to muscle contraction. In the case of a PT transection, the retraction can be visualized and monitored radiographically with a sagittal X-ray image by the retraction of the PB (*patella alta* [6, 24, 25]). Surgical restoration of human patellar tendon ruptures requires a cerclage to divert forces that cause proximilization [6, 26].

Similarly, this murine study has shown that without augmentation, the muscular retraction of the quadriceps causes elongation and subsequent gap formation and/or graft failure (NA-group, Additional file 1). A simple circumferential transfascial cerclage around the PT (TFSC), as often used with thick sutures or wires in humans and large animals [6, 27, 28], potentially can counterforce tension through the quadriceps muscle. However, in the mouse model, such a suture cannot hold its position and either ruptures or slips along the patellar bone. In the first case, the cerclage suture is not strong enough, in the latter the thin tendon and fascia layers do not allow a secured anchoring of the cerclage. Ultimately, such a circumferential cerclage causes graft elongation and ultimately graft failure (69% of cases).

As shown in the DCA model, transosseous fixation of the cerclage through the patellar bone is essential in the murine knee. Using this double cerclage technique, a significant preservation of the patella position is achieved over the time of 8 weeks, shown in an equal patello-tibial distance (PTD) to the contralateral tendons. During this critical time period (proliferation phase), the graft has time to integrate into the defect [29]. Due to the angled physiological movement of the patella bone in the knee joint, the proximalization as a result of the quadriceps force does not occur in a collinear manner [30]. Therefore, the radiographical grading system was introduced to provide more information about the functional result of the patella-graft-complex. While there are other imaging techniques that could be used for evaluation, including MRI or ultrasound, X-ray was chosen because of its ease of accessibility, low cost, and user-independent strengths. A flat plane of the graft anterior to the trochlear groove is desired otherwise the graft is exposed to additional shear and bending forces once the graft is pulled proximal to the groove. For standardization reasons, the supracondylar spur was chosen as a reference point for grading. Ninety degree flexion is a representative state of the murine knee during the entire gait cycle and was therefore chosen as a fixed position for standardized radiographs [31]. In such a setting, and with the posterior cruciate ligament being intact [32], PBP grade I and II ensure a flat plane of the graft, as the distal pole of the PB is anterograde to the condyle edge and the graft does not bend over the condyle curvature (Fig. 2b, c). Grade III is borderline and at grade IV and V the graft must be bent (Figs. 2d–f). The presented model meets these demands of a flat graft plane for linear tendon integration. Both PTD and PBP were marginally smaller compared to the contralateral at the early time point (4 weeks) due to intraoperative overdistalization of 15–20% of the PB; a necessity to allow the graft to have stump contact from the very beginning, avoiding iatrogenic gap formation. However, the distance gained by the PTD and PBP over time reflects tensile forces being transmitted through the graft. As tensile loading stimulates tendon-specific genes and tenogenic precursor cells, this effect is desired [19, 20, 33].

Stability is largely provided by the TPC, which is corroborated by a correlation between the failure of the TPC and PBP grading as well as TPC failure and gap formation. A decent number of grafts showed a gap filled with scar up to 1 mm between the graft and one of the tendon stumps. This might be due to graft shrinkage or the nominal graft space elongation. The original PT is cut off the bone very closely in order to gain as long of graft as possible; therefore, the short tendon stump leaves no substance for a sufficient end-to-end anastomosis. A limitation of a small transplantation model is that a seamless adaptation of the graft is not achievable with suturing at that scale; therefore, the graft is kept in place with two adaptation sutures to the corresponding bones. In addition to the TPC, the application of a TMC is required. The TMC frames the whole PB and inserts in the muscle, thus more proximal than the TPC. From our experience, the TMC prevents lateral patellar dislocation by moving the soft musculous pivot point further proximal. In future studies, the two cerclages can potentially be transected to terminate the bypass structure and allow a full load onto the graft.

From the technical point of view, our experience has shown that adequate application of the patello-tibial-cerclage suture (PTC) is the essential step of surgery. A frame structure along the physiological plane of the PT is biomechanically the most stable method but requires drilling a hole into the PB in the coronal plane of the PT [6, 26]. Different drill sizes, cerclage materials, and cerclage beading techniques were tested (data not shown). Given the thickness of the mouse PB at 16 weeks of age is 0.8 ± 0.07[0.7–0.9] mm and the required drill hole length averages 1 mm. In our experience, a 0.2 mm is the ideal drill bit size for achieving the desired drill hole through the mouse patellar bone. The entry point needs to be exactly at mid-thickness of the PB, the bicortical drilling needs to be strictly axial with a precisely aligned drill bit axis; otherwise, the drill tip will rotate conically and cause undesired widening of the drill hole. Numerous (0.08–0.15 mm) titanium and nitinol wire cerclages were also tested, but they all led to cerclage failures (data not shown). The cerclage pull-through is a critical step as the beading needle might damage the very thin posterior or anterior cortical wall (see Additional file 3). Taking those circumstances into account, the total amount of surgery-related complications was still 20%. For future implications, computer-assisted and navigated drilling and beading might lead to lower failure rates but might prolong surgery time.

Although a repeatable surgical method with reproducible results was presented, there are certain limitations to this study. First, surgical success was assessed based on anatomical and functional outcomes only. However, a strongly retracted patellar position as macroscopically and radiologically shown in the two NCA and TFSC techniques creates dysfunctional physiology. Application of these surgical techniques to interrogate any specific hypotheses should consider evaluating the biomechanical integrity of the healing tissue.

In the DCA, all animals returned to a physiological movement and loading pattern within the study time, demonstrating a physiological function. To our knowledge, this is the first study to show an anatomical position of the patellar bone over the time period of 8 weeks. Another limitation is the necessity of the ideal surgery conditions like the required standardization fixture, extensive suture handling under sterile conditions and long anesthetic and surgical times. This certainly is a limitation of all in-vivo trials and future work should address optimization of these specific conditions.

## Conclusions

In summary, this study has shown that a sufficient cerclage, acting as a tensile bypass of the quadriceps muscle force on the tibia, is crucial in order to avoid elongation of the bone-graft-bone-complex. The necessity of a bone-to bone cerclage for patellar graft implantation was shown for a mouse model. A surgical technique was presented that takes these considerations into account. As compared to previous single cerclage techniques used in both murine models and as treatments in human medicine, it was shown that a two cerclage technique gains superior consistency and thus is a more efficient technique for data collection. This technique can be utilized for future experimental models, streamlining data collection in context of patellar tendon healing and regeneration processes. In particular, this can be helpful for transplantations of allo- and autografts, or models involving implantation of xenografts or tissue-engineered matrices.

## Supplementary information

**Additional file 1.** Preliminary murine patellar tendon transplantation model without augmentation (no-cerclage-augmentation group (NCA)). Surgical procedure, macroscopic, and radiographic evaluation of the NCA model.

**Additional file 2.** Preliminary murine patellar tendon transplantation model with transfascial suture cerclage (TFSC group). Surgical procedure, macroscopic, and radiographic evaluation of the TFSC model.

## Data Availability

The datasets used and/or analyzed during the current study are available from the corresponding author on reasonable request.

## Abbreviations

DCA: Dual-cerclage-augmentation
NCA: No-cerclage-augmentation
PBP: Patella bone position grading system
PT: Patellar tendon
PTD: Patello-tibial distance
PTG: Patellar tendon graft
TFSC: Transfascial suture cerclage
TMC: Tibio-musculotendinous cerclage
TPC: Tibio-patellar cerclage
