# Supplementary material for: Murine patellar tendon transplantation requires transosseous cerclage augmentation — development of a transplantation model for investigation of systemic and local drivers to healing
Source: J Orthop Surg Res. 2019 Dec 2;14:410. doi: 10.1186/s13018-019-1475-4 (PMC6889740; doi:10.1186/s13018-019-1475-4)
Supplement: Supplementary file 2 — Additional file 1. Preliminary murine patellar tendon transplantation model without augmentation (no-cerclage-augmentation group (NCA)). Surgical procedure, macroscopic, and radiographic evaluation of the NCA model. [file 13018_2019_1475_MOESM1_ESM.docx]

**Supplement 1:**

**Preliminary murine patellar tendon transplantation model without augmentation (no-cerclage-augmentation group (NCA))**

Surgical Procedure

Surgery was performed on six C57BL/6J mice following a similar procedure as described in the main manuscript with the exception of the application of a cerclage. After the tendon was lifted, it was stitched back through the lateral retinaculum, around the tendon stumps and knotted at the medial retinaculum, using 9-0 Polypropylene suture (P90062; Visionary Medical Supplies, Madison, WI; see Figure S1-1). Previous attempts on cadavers with thicker needles or sutures resulted in immediate tear out or tendon rupture. Final steps and postoperative care were performed as described in the manuscript.

Macroscopic and radiographic evaluation


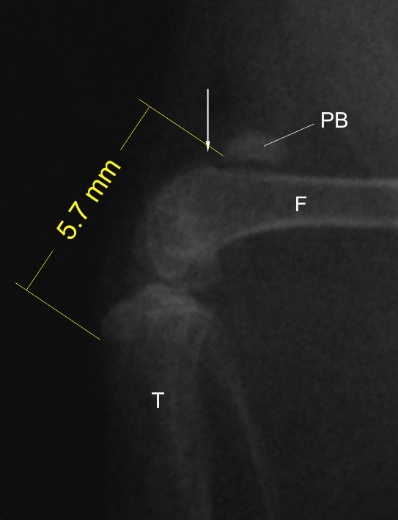

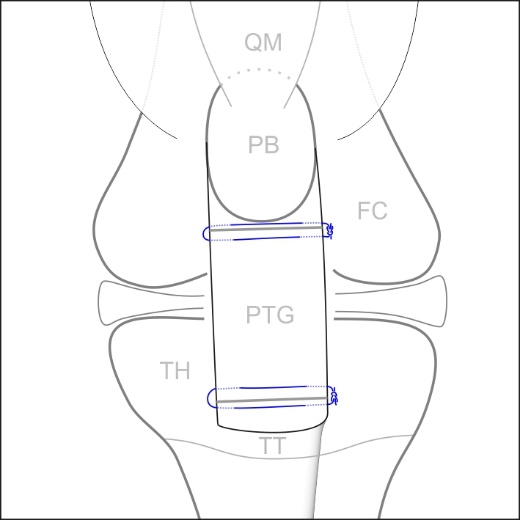
Mice were sacrificed 5-7 days post surgery. Macroscopically, all specimen showed a proximalized patella as a result of implantation failure. Proximal suture failure was observed in one animal, distal suture failure in two, and tendon graft rupture or fraying in three animals (see Figure S1-3). Sagittal radiographs of all 6 specimen showed an extremely proximalized patella (patella position 5; see Figure S1-2).

**Fig. S1-1**

**Fig. S1-2**

**Fig. S1-1:** Schematic of murine patellar tendon transplantation without augment-tation: The patellar tendon graft (PTG) is fixed to the tendon stumps using a 9-0 Sutralene suture (blue). PB = patellar bone, TT = tibial tuberosity, TH = tibial head, FC = femur condyles, QM = quadriceps muscle.

**Fig. S1-2:** Representative sagittal radio-graph shows a retracted patellar bone (PB) proximally to the supraconylar spur (arrow) with a patella-tibial-tuberosity-distance of 5.7 mm. T = tibia, F = femur.


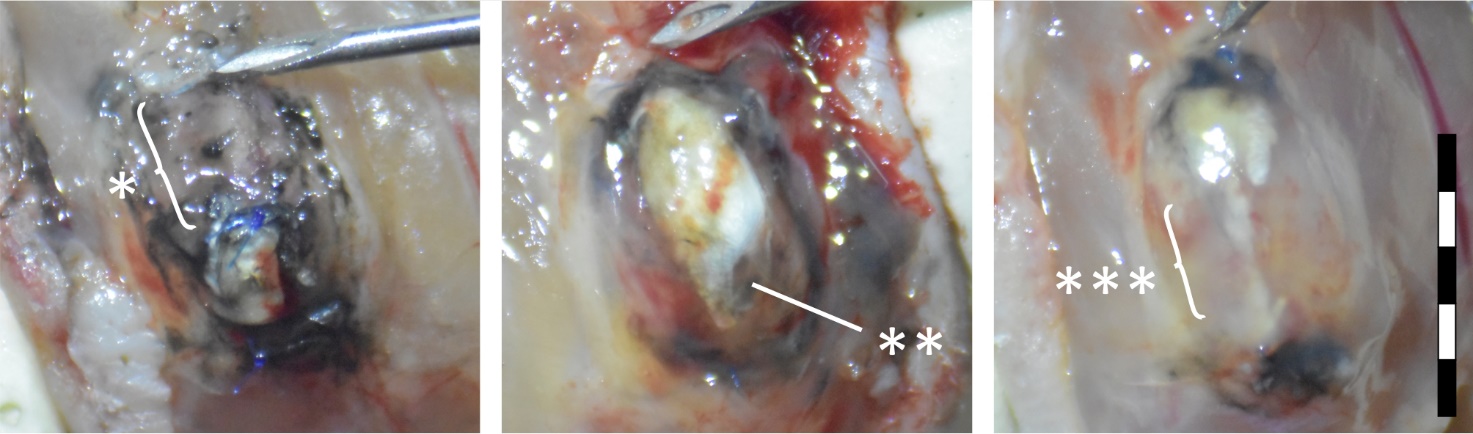


**Fig. S1-3:** Macroscopic images, 5-7 days post transplantation, showing transplant failure. **(a)** The graft tore out proximally, leaving a 2.5 mm gap (*) between proximal tendon stump (indicated by needle tip) and proximal graft stump (blue sutures). **(b)** The tendon elongated and subsequently tore out distally (**). **(c)** A case in which both sutures were sufficien but the tendon graft elongated (***) and frayed out. In this model india ink was used to mark the tendon stumps (black pigmentation). Scale = 5 mm.

**(c)**

**(b)**

**(a)**
