# Supplementary material for: Murine patellar tendon transplantation requires transosseous cerclage augmentation — development of a transplantation model for investigation of systemic and local drivers to healing
Source: J Orthop Surg Res. 2019 Dec 2;14:410. doi: 10.1186/s13018-019-1475-4 (PMC6889740; doi:10.1186/s13018-019-1475-4)
Supplement: Supplementary file 3 — Additional file 2. Preliminary murine patellar tendon transplantation model with transfascial suture cerclage (TFSC group). Surgical procedure, macroscopic, and radiographic evaluation of the TFSC model. [file 13018_2019_1475_MOESM2_ESM.docx]

**Supplement 2:**

**Preliminary murine patellar tendon transplantation model with transfascial suture cerclage (TFSC group)**

Surgical Procedure

Surgery was performed on 16 C57BL/6J and 16 MRL/MpJ mice. 8 mice of each strain were randomly assigned to 2 different survival time-points: 28 days (4w) and 56 days (8w). Half of the animals of each strain were used for autografts and half for allografts. Procedure steps were similar to those described in the main manuscript with the exception of application of the cerclage. After drilling the tunnel underneath the tibial tuberosity, a 5-0 Ethibond Excel suture was stitched around the tibia by transfixating the musculotendinous junctions of the vastus medialis, rectus femoris and vastus lateralis muscles and pulled through the tunnel (see Figure S2-1). Subsequently, tendon explanation was performed as described in the manuscript. The tendon graft was stitched back into the corresponding animal, using the 9-0 Polypropylene suture as shown in figure S2-1. After tendon graft replantation, the 6-0 cerclage suture was pulled until gaps on both sides of the graft were closed and the cerclage suture knotted. Final steps and postoperative care were performed as described in the manuscript. Mice were sacrificed 4 or 8 weeks post surgery.

Macroscopic and radiographic evaluation

Macroscopically, a significant number of animals showed cerclage failure. In 11 animals (34%), the cerclage was torn. In another 11 animals (34%) the tendon “slipped” distally over the patellar bone, causing a partial or total delamination of the quadriceps tendon insertion (see figure S2-2). Results of the macroscopic and radiographic assessments are shown in table S2.


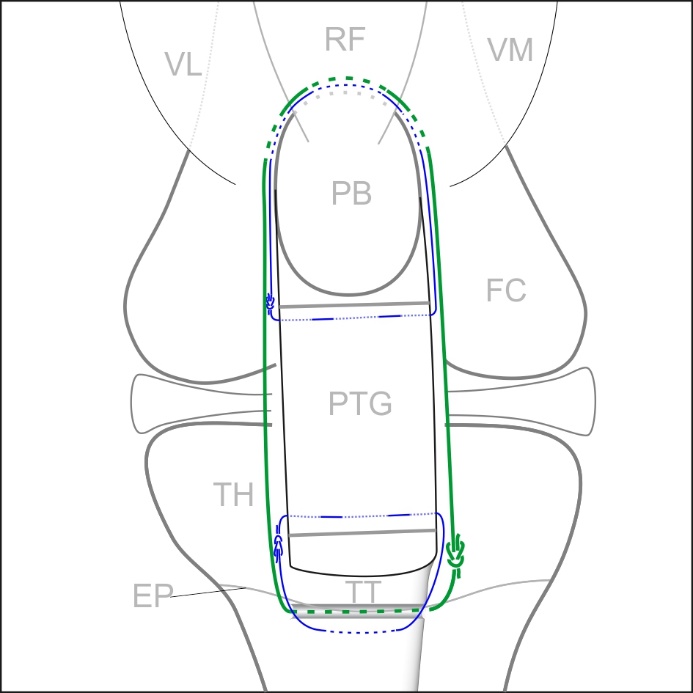


**Fig. S2-1:** Schematic of patellar tendon transplantation with circumferential cerclage augmentation: The cerclage suture (green) is intended to keep the tibio-patellar distance consistent, while the 9-0 Polypropylene suture (blue) holds the graft (PTG) in place. PB=patellar bone, VL=vastus lateralis, RF=rectus femoris, VM=vastus medialis, FC=femoral condyles, TT=tibial tuberosity, TH=tibial head, EP=epiphyseal plate.

**Fig. S2**

**Fig. S2-2:** Macroscopic results at 4 (112, 77, 61 - a,b,c) and 8 (a,b,c) weeks after surgery: **a)** In 34% of cases the cerclage suture slipped ventrally over the patellar bone and delaminated the quad muscle insertion. The delaminated retracted patellar bone appears very red, as a sign of irrigation. The proximal loop of the cerclage is visible below the distal part of the patellar bone. The tendon graft, (indicated) appears contracted, due to loss of proximal fixation. **b)** The cerclage suture tore out distally, at its curve around the tibial drill edge, causing patellar bone retraction (patellar bone position indicated). The tendon graft is macroscopically not distinguishable from the surrounding scar tissue formation. **c)** In a few cases the cerclage suture tore proximally. In this case, the gap between the visible graft (*) and patellar bone is approximately 2-3 mm wide (indicated). Scale = 5 mm.

Scale = 5 mm.


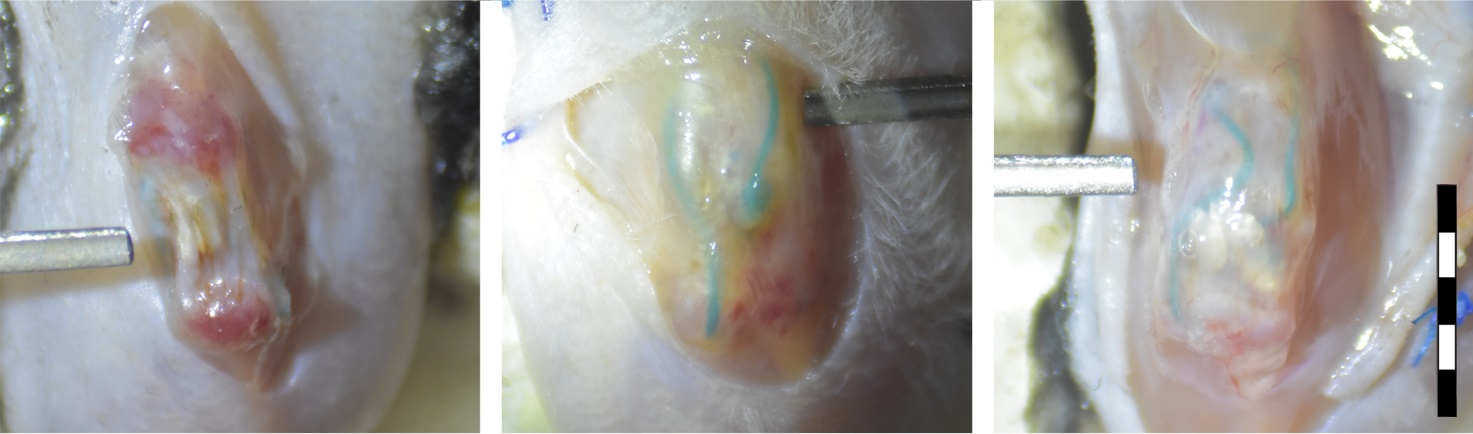


*****

**Fig. S2-2 (a)**

**(c)**

**(b)**

**Fig. S2-1**

|  | Total  [n] | Proximal cerlcage rupture  [n] | Distal cerlcage rupture  [n] | Paratendonal cerlcage rupture  [n] | Proximal cerlage slippage  [n] | Total cerlage failures  [n] | Graft length^#$^  [mm] | Patellar bone position^#$^  [grade] |
| --- | --- | --- | --- | --- | --- | --- | --- | --- |
| 4w C57BL/6J host | 8 | 2 | 1 | 1 | 2 | 6 (19%) | 4.6 ± 0.4 | 3.8 ± 1.2* |
| 4w MRL/MpJ host | 9 |  | 2 |  | 3 | 5 (16%) | 4.4 ± 0.33 | 2.3 ± 0.66* |
| 8w C57BL/6J host | 8 |  | 1 | 1 | 2 | 4 (13%) | 4.5 ± 0.41 | 3.8 ± 1.1 |
| 8w MRL/MpJ host | 7 |  | 3 |  | 4 | 7 (22%) | 4.7 ± 0.33 | 2.8 ± 0.81 |
| Total | 32 | 2 | 7 | 2 | 11 | 22 (69%) | 4.6 ± 0.37 | 3.1 ± 1.1 |

**Table S2-1** Cerclage failure was defined as positive when the cerclage ruptured or slipped over the patellar bone and ended in a position distal or ventral of the visible portion of the patellar bone. ^#^ Graft length and patellar bone position were obtained radiologically as described in the manuscript. ^$^ No significance differences were found within groups of the same sacrifice time point. *Only difference found between the two strains at 4 weeks after surgery (p<0.05).
